# Genetic architecture of body weight, carcass, and internal organs traits of Ghanaian local chickens

**DOI:** 10.3389/fgene.2024.1297034

**Published:** 2024-03-13

**Authors:** Roland A. Kanlisi, Esinam N. Amuzu-Aweh, Augustine Naazie, Hope R. Otsyina, Terra R. Kelly, Rodrigo A. Gallardo, Susan J. Lamont, Huaijun Zhou, Jack Dekkers, Boniface B. Kayang

**Affiliations:** ^1^ Department of Animal Science, University of Ghana, Accra, Ghana; ^2^ United States Agency for International Development Feed the Future Innovation Lab for Genomics to Improve Poultry, University of California, Davis, CA, United States; ^3^ School of Veterinary Medicine, University of Ghana, Accra, Ghana; ^4^ One Health Institute, University of California, Davis, CA, United States; ^5^ School of Veterinary Medicine, University of California, Davis, CA, United States; ^6^ Department of Animal Science, Iowa State University, Ames, IA, United States; ^7^ Department of Animal Science, University of California, Davis, CA, United States

**Keywords:** GWAS, Ghanaian chicken ecotypes, carcass traits, internal organ traits, growth traits

## Abstract

Information on the genetic architecture of the production traits of indigenous African chicken is limited. We performed a genome-wide association study using imputed Affymetrix Axiom^®^ 600K SNP-chip genotypes on 1,113 chickens from three agroecological zones of Ghana. After quality control, a total of 382,240 SNPs remained. Variance components and heritabilities for some growth, carcass and internal organ traits were estimated. The genetic and phenotypic correlations among these traits were also estimated. The estimated heritabilities of body weight at week 22 (BW22), average daily gain (ADG), dressed weight, breast weight, thigh weight, wing weight, drumstick weight, and neck weight were high and ranged from 0.50 to 0.69. Estimates of heritabilities for head weight, shank weight, and gizzard weight were moderate (0.31–0.35) while those of liver weight, back weight, dressing percentage, and heart weight were low (0.13–0.21). The estimated heritabilities of dressed weight, breast weight, wing weight, drumstick weight, neck weight, shank weight, and gizzard weight, corrected for BW22, were moderate (0.29–0.38), while the remaining traits had low heritability estimates (0.13–0.21). A total of 58 1-Mb SNP windows on chromosomes 1, 2, 4, 5, 6, 7, 8, 9, 13, 18, and 33 each explained more than 1% of the genetic variance for at least one of these traits. These genomic regions contained many genes previously reported to have effects on growth, carcass, and internal organ traits of chickens, including *EMX2, CALCUL1, ACVR1B, CACNB1, RB1*, *MLNR, FOXO1, NCARPG, LCORL, LAP3, LDB2*, *KPNA3,* and *CAB39L*. The moderate to high heritability estimates and high positive genetic correlations suggest that BW22, ADG, dressed weight, breast weight, thigh weight, wing weight, drumstick weight, and neck weight could be improved through selective breeding.

## 1 Introduction

Several indigenous African chicken ecotypes, including the Forest (FO), Interior savanna (IS) and Costal savanna (CS) ecotypes of Ghana have been reported (Walugembe et al., 2020). These chickens are hardy and thrive quite well in severe climates and environments (Pius et al., 2021). They are a major source of protein and play very important roles in sustaining the livelihoods of many households in Africa. Furthermore, there is also a perception that the meat of indigenous chicken ecotypes is very tasty, thus contributing in a large part, to a high demand for the meat of indigenous chickens in Ghana and many other parts of Africa (Asante-Addo and Weible, 2020; Ragasa et al., 2020).

The ability of indigenous chickens to thrive in different agroecological zones of Africa can in part be attributed to the variety of adaptive traits they possess, including thermotolerance, ability of escape predation, resistance to several endemic diseases (Mpenda et al., 2019), and a capacity to thrive under conditions of feed and water scarcity. Notwithstanding these important adaptive traits, indigenous African chicken ecotypes tend to have comparatively lower growth rates and body sizes (Munisi et al., 2015; Birteeb et al., 2016). As a result, many subsistence farmers tend to breed them with other breeds of chicken with the objective of increasing their body weights, a situation that can occasion the loss of their adaptive traits.

Some studies on the production traits of indigenous African chicken are available (Osei-Amponsah et al., 2013; Dekkers et al., 2018) but very few comprehensive genome-wide association studies (GWAS) on their production traits have been carried out. It is therefore imperative to unravel the genetic architecture of the production traits of indigenous chicken populations of Ghana to provide better insights for the genetic improvement of these traits in future. This GWAS therefore sought to examine the genetic architecture of the growth, carcass, and internal organ traits of the Forest, Interior and Coastal Savanna chicken ecotypes of Ghana.

## 2 Materials and methods

### 2.1 Experimental design

A total of 1,113 chickens, made up of the CS, the IS, and FO chicken ecotypes were used in this study. These are chickens whose parents have been described in Walugembe et al., 2020. Each ecotype was housed separately in deep litter pens. The dimensions of each pen were 2.54 m × 2.2 m × 2.2 m and housed a maximum of 40 birds. From day 1 to week 8, all birds were fed a standard chick starter mash, while from week 9 to week 22 they were fed a standard chick grower mash. Water was available on an *ad libitum* basis. Vaccination, feeding, and all other management practices were the same for all the chickens in the study.

At hatch, the body weight of every bird was measured and thereafter measured fortnightly until 22 weeks of age. From this data, average daily gain (ADG) was calculated as the linear regression of body weight on days of age. At week 23, the birds were euthanized and several carcass and internal organ traits including breast, thigh, wing, drumstick, neck, back, shank, head, gizzard, heart, liver and dressed weights were measured. Except for the gizzard, heart, and liver, the rest of the parts contained some skin.

### 2.2 Genotyping

Blood samples were collected from the wing veins of the chicks at 5 weeks of age using Whatman FTA cards (Sigma-Aldrich, St. Louis, MO, United States). Genomic DNA was isolated from the FTA cards for genotyping by sequencing (GBS) using a 5K GBS panel which was developed specifically for local Ghanaian and Tanzanian chicken ecotypes. A total of 5,238 SNPs were included in the SNP panel. Details on the development of the GBS panel are given in Walugembe et al. (2022). The genome sequences obtained were subjected to a customized SNP-pipeline that resulted in 5K SNP genotypes of each bird. These genotypes were then imputed to 382,240 SNPs that remained after quality control of high-density genotype data of relatives using Affymetrix Axiom^®^ 600K SNP chip [the high-density genotype data are described in Walugembe et al. (2020)]. Imputation was performed using Fimpute (Sargolzaei et al., 2014).

### 2.3 Population structure

The FO, CS, and IS chicken ecotypes of Ghana that were used in this study are reported to originate from three ancestral populations (Walugembe et al., 2020). To deduce the proportion of ancestral subpopulations in each chicken, we carried out admixture analyses on the imputed genotypes using the Admixture software (Alexander et al., 2009), with the number of sub-populations set to three. These ancestral subpopulation proportions were used as covariates in the downstream genetic analyses.

### 2.4 Genetic parameters

Variance components and heritabilities were estimated using the following univariate linear model: $y=\text{Xb}+Z_{a}a+e,$ (Model 1), where **y** is the vector of phenotypes (Body weight at week 22, ADG, breast weight, drumstick weight, thigh weight, wing weight, dressed weight, dressing percentage, head weight, neck weight, shank weight, back weight, gizzard weight, liver weight, and heart weight); **b** is the vector of the fixed effects (replicate, sex, and pen by replicate), and covariates (three ancestral subpopulation proportions obtained from the admixture analysis); ***a*** is the vector for random animal genetic effect; ***e*** is the residual effect; **X** and **Z**_***a***_ are the incidence matrices for the effects in the ***b*** and ***a*** vectors respectively.

Body weight at week 22 (BW22) was also fixed as a covariate (Model 2) for some of the traits, i.e., breast, thigh, wing, drumstick, neck, back, shank, head, gizzard, heart, liver and dressed weight. The covariate explains out some of the variation in these traits due to body weight.

The genetic and phenotypic correlations between traits were estimated by fitting pairwise bivariate models with the same effects as in the univariate linear models. All models were implemented in ASReml 4 (Gilmour et al., 2015).

### 2.5 Genome-wide association and bioinformatics analyses

Genome-wide association analysis was performed using Bayes B (Meuwissen et al., 2001; Cheng et al., 2015), as implemented in the JWAS package (Cheng et al., 2015), to estimate the genetic variance accounted for by each 1-megabase (Mb) SNP window across Gallus *gallus* 6 genome build. Both Models 1 and 2 were used. 1-Mb SNP regions that explained more than 1% of the genetic variance in a trait were considered significant. To identify genes within significant 1-Mb SNP windows, we resorted to the Genome Data Viewer in NCBI—https://www.ncbi.nlm.nih.gov/genome/gdv/browser/genome/?id=GCF_000002315.6.

## 3 Results

### 3.1 Population structure

**FIGURE 1:**
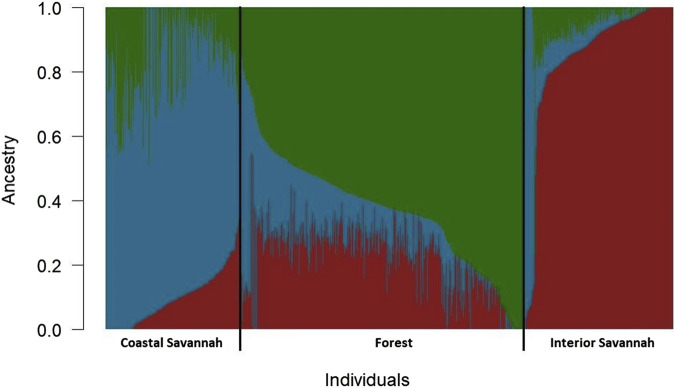
Admixture plot showing mixed ancestry among birds from the three Ghanaian chicken ecotypes.

The admixture analysis based on identity by state as shown in Figure 1, indicates that notwithstanding the evidence of admixture, all the three ecotypes appeared to have come from three distinct ancestral populations. The IS ecotype had a high proportion of subpopulation 1 (0.83) but with lower proportions of subpopulations 2 (0.11) and 3 (0.05). The CS ecotype had a higher proportion of subpopulation 2 (0.74) and lower proportions of subpopulations 1 (0.09) and 3 (0.16), while the FO ecotype had a higher proportion of subpopulation 3 (0.63) and lower proportions of subpopulation 1 (0.22) and subpopulation 2 (0.15).

### 3.2 Genetic parameters

The heritabilities and correlations of the growth, carcass, and internal organ traits of the FO, CS, and IS chicken ecotypes of Ghana, i.e., body weight at 22 weeks of age (BW22), average daily gain (ADG), breast weight (BrW), drumstick weight (DW), thigh weight (TW), wing weight (WW), dressed weight (DrW), and dressing percentage (DP) were estimated. These are presented in Tables 1, 2, while the estimated heritabilities and correlations of other body parts and internal organs, i.e., head weight (HW), neck weight (NeW), shank weight (ShW), back weight (BaW), gizzard weight (GzW), liver weight (LiW), and heart weight (HeW), are presented in Tables 3, 4.

**TABLE 1:** Estimates of heritabilities (along diagonal) and of genetic (above diagonal) and phenotypic (below diagonal) correlations for growth and carcass traits from Model 1 (without BW22 as a covariate).

Trait	BW22	ADG	DrW	DP	BrW	TW	WW	DW
BW22	**0.58** (0.07)	0.96 (0.01)	0.95 (0.02)	0.24 (0.17)	0.86 (0.04)	0.92 (0.03)	0.88 (0.03)	0.88 (0.03)
ADG	0.90 (0.006)	**0.69** (0.07)	0.91 (0.02)	0.22 (0.16)	0.79 (0.04)	0.86 (0.04)	0.83 (0.04)	0.82 (0.04)
DrW	0.87 (0.0008)	0.82 (0.01)	**0.60** (0.07)	0.53 (0.13)	0.94 (0.02)	0.95 (0.02)	0.93 (0.02)	0.91 (0.02)
DP	0.004 (0.03)	0.06 (0.03)	0.41 (0.03)	**0.15** (0.06)	0.61 (0.13)	0.47 (0.16)	0.44 (0.15)	0.38 (0.16)
BrW	0.72 (0.02)	0.67 (0.02)	0.87 (0.008)	0.43 (0.03)	**0.52** (0.07)	0.84 (0.0.5)	0.86 (0.04)	0.79 (0.05)
TW	0.78 (0.01)	0.73 (0.02)	0.82 (0.01)	0.27 (0.03)	0.65 (0.02)	**0.51** (0.07)	0.89 (0.04)	0.94 (0.03)
WW	0.78 (0.01)	0.73 (0.02)	0.87 (0.009)	0.30 (0.03)	0.73 (0.02)	0.73 (0.02)	**0.62** (0.07)	0.94 (0.03)
DW	0.82 (0.01)	0.71 (0.02)	0.85 (0.009)	0.35 (0.03)	0.68 (0.02)	0.72 (0.02)	0.80 (0.01)	**0.55** (0.07)

BW22, Body weight at 22 weeks of age; ADG, average daily gain; BrW, breast weight; DW, drumstick weight; TW, thigh weight; WW, wing weight; DrW, Dressed weight and DP, Dressing percentage. Standard errors are in parenthesis.

**TABLE 2:** Estimates of heritabilities (along diagonal) and of genetic (above diagonal) and phenotypic (below diagonal) correlations for growth and carcass traits from Model 2 (with BW22 covariate).

Trait	DrW	BrW	TW	WW	DW
DrW	**0.30 (0.07)**	0.77 (0.08)	0.75 (0.1)	0.71 (0.09)	0.61 (011)
BrW	0.74 (0.01)	**0.29 (0.07)**	0.39 (0.17)	0.48 (0.13)	0.22 (0.18)
TW	0.58 (0.02)	0.30 (0.03)	**0.21 (0.07)**	0.58 (0.14)	0.76 (0.12)
WW	0.67 (0.02)	0.45 (0.03)	0.43 (0.03)	**0.38 (0.08)**	0.75 (0.09)
DW	0.67 (0.02)	0.35 (0.03)	0.42 (0.03)	0.57 (0.02)	**0.29 (0.07)**

BW22, Body weight at 22 weeks of age; BrW, breast weight; DW, drumstick weight; TW, thigh weight; WW, wing weight; and DrW, Dressed weight. Standard errors are in parenthesis.

Bold values represent Estimates of Heritabilities.

**TABLE 3:** Estimates of heritabilities (along diagonal) and of genetic (above diagonal) and phenotypic (below diagonal) correlations for carcass traits and internal organs from Model 1 (without BW22 as a covariate).

Trait	BaW	HW	NeW	ShW	GzW	LiW	HeW
BaW	**0.17 (0.06)**	0.35 (0.03)	0.51 (0.02)	0.45 (0.03)	0.36 (0.03)	0.39 (0.03)	0.53 (0.02)
HW	0.72 (0.1)	**0.31 (0.07)**	0.76 (0.08)	0.67 (0.09)	0.34 (0.13)	0.35 (0.17)	0.61 (0.11)
NeW	0.89 (0.06)	0.57 (0.02)	**0.50 (0.07)**	0.67 (0.07)	0.48 (0.1)	0.60 (0.12)	077 (0.07)
ShW	0.62 (0.09)	0.44 (0.03)	0.58 (0.02)	**0.34 (0.07)**	0.47 (0.07)	0.58 (0.12)	0.65 (0.09)
GzW	0.54 (0.1)	0.15 (0.03)	0.34 (0.03)	0.66 (0.08)	**0.35 (0.07)**	0.75 (0.1)	0.63 (0.1)
LiW	0.58 (0.12)	0.16 (0.03)	0.33 (0.03)	0.36 (0.08)	0.37 (0.03)	**0.13 (0.05)**	0.66 (0.12)
HeW	0.77 (0.08)	0.45 (0.03)	0.52 (0.02)	0.39 (0.03)	0.25 (0.03)	0.35 (0.03)	**0.21 (0.06)**

HW, head weight; NeW, neck weight; ShW, shank weight; GzW, gizzard weight; LiW, liver weight; HeW, heart weight; BaW, Back weight. Standard errors are in parenthesis.

**TABLE 4:** Estimates of heritabilities (along diagonal) and of genetic (above diagonal) and phenotypic (below diagonal) correlations for carcass traits and internal organs from Model 2 (with BW22 as a covariate).

Trait	BaW	HW	NeW	ShW	GzW	LiW	HeW
**BaW**	**0.17 (0.06)**	0.08 (0.03)	0.14 (0.03)	0.03 (0.03)	0.08 (0.03)	0.11 (0.03)	0.24 (0.03)
**HW**	0.32 (0.24)	**0.20 (0.06)**	0.47 (0.16)	0.28 (0.18)	−0.20 (0.19)	−0.37 (0.26)	0.15 (0.21)
**NeW**	0.65 (0.21)<	0.43 (0.03)	**0.29 (0.07)**	0.09 (0.17)	−0.10 (0.17)	−0.08 (0.24)	0.33 (0.18)
**ShW**	−0.27 (0.21)	0.25 (0.03)	0.30 (0.03)	**0.34 (0.07)**	0.33 (0.14)	−0.03 (0.22)	0.07 (0.18)
**GzW**	−0.15 (0.21)	−0.05 (0.03)	0.09 (0.03)	0.24 (0.03)	**0.35 (0.07)**	0.47 (0.18)	0.25 (0.18)
**LiW**	−0.38 (0.31)	−0.05 (0.03)	0.06 (0.03)	0.12 (0.03)	0.21 (0.03)	**0.13 (0.05)**	0.13 (0.25)
**HeW**	0.19 (0.2)	0.28 (0.03)	0.26 (0.03)	0.09 (0.03)	0.01 (0.03)	0.14 (0.03)	**0.21 (0.06)**

HW, head weight; NeW, neck weight; ShW, shank weight; GzW, gizzard weight; LiW, liver weight; HeW, heart weight; BaW, Back weight. Standard errors are in parenthesis.

For Model 1 (without BW22 as a covariate), as shown in Tables 1, 3, the estimated heritabilities of DP, LiW, HeW and BaW were low (0.13–0.21), while those for the other traits ranged from medium to high (0.31–0.69). Estimates of the genetic correlation between DP and BW22 and between DP and ADG were low. DP also had low phenotypic correlations with BW22, ADG, and TW. HW had low phenotypic correlations with GzW and LiW, while GzW also had a weak correlation with HeW. The rest of the traits had positive medium to high genetic and phenotypic correlations with each other.

For Model 2 (with BW22 as a covariate), as shown in Tables 2, 4, the estimated heritabilities for TW, LiW, and BaW were low (0.13–0.21), while the estimated heritabilities for some other traits were moderate and ranged from 0.29 for BrW to 0.38 for WW. GzW had negative estimates of genetic correlations with HW and NeW. LiW also had negative genetic correlation estimates with HW, NeW and ShW. The phenotypic correlation between BaW and ShW, and between GzW and LiW was negative. In addition, HW also had negative phenotypic correlations with GzW and LiW. Among the traits, ShW and NeW, BaW and NeW, GzW and LiW, and HeW, and NeW had high genetic correlation estimates, while HW and NeW, and HW, and BaW had high phenotypic correlations. The rest of the traits had low estimates of genetic and phenotypic correlations.

The effects of the ancestral subpopulation proportions as covariates in the downstream genetic analysis were statistically not significant.

### 3.3 Genome-wide association study

After quality control, a total of 1,113 birds and 382,240 SNPs were used for the GWAS. The percentage of genetic variance explained by 1-Mb genomic regions that are associated with the growth, carcass, and internal organ traits, with and without BW22 as covariate, are shown in Table 5 and the genes that are within significant 1-Mb windows are shown in Tables 6, 7.

**TABLE 5:** Percentage of genetic variance explained by 1-Mb genomic regions that are associated with growth, carcass, and internal organ traits (≥1.0% of genetic variance) based on the Bayes-B method, using Model 1 (without BW22 as covariate) and Model 2 (with BW22 as covariate).

Trait	Chr	1-Mb window	No. of markers in window	% Genetic variance explained by window
Model 1 (without BW22 as covariate)				
BW22	1	169–170	383	1.4
	1	170–171	345	10.4
	1	171–172	376	2.1
	2	22–23	305	2.7
	2	39–40	319	1.3
	2	110–111	290	1.1
	4	75–76	263	19.7
	5	27–28	410	1.2
ADG	1	133–134	357	1.1
	1	170–171	345	9.6
	1	171–172	376	1.0
	4	75–76	263	11.6
	33	3.2–3.3	17	1.0
DrW	1	170–171	345	2.5
	1	171–172	376	3.7
	4	75–76	263	14.0
	18	7.0–8.0	713	1.9
BrW	1	18–19	312	1.0
	1	170–171	345	1.7
	1	171–172	376	1.4
	1	180–181	372	1.0
	1	182–183	404	2.1
	4	75–76	263	12.2
	33	3.2–3.3	17	1.1
DW	1	170–171	345	11.9
	4	75–76	263	20.6
TW	1	169–170	383	1.0
	1	170–171	345	9.8
	1	171–172	376	1.4
	4	75–76	263	19.6
WW	1	170–171	345	12.8
	1	171–172	376	1.2
	1	195–196	404	1.7
	4	75–76	263	25.2
BaW	1	171–172	376	4.6
	4	66–67	358	1.0
	4	75–76	263	5.3
HW	1	170–171	345	1.9
	4	75–76	263	1.4
	8	27–28	479	2.2
NeW	1	12–13	438	1.1
	1	170–171	345	5.2
	4	75–76	263	6.4
	9	2.0–3.0	524	1.9
ShW	1	164–165	346	1.2
	1	170–171	345	9.4
	4	75–76	263	33.9
GzW	1	170–171	345	7.4
	4	75–76	263	15.0
	4	22–23	288	1.3
LiW	4	75–76	263	20.5
HeW	1	155–156	370	1.1
	1	170–171	345	2.1
	4	75–76	263	2.7
	6	30–31	444	1.2
	7	18–19	440	2.2
	13	6.0–7.0	471	1.1
	33	3.2–3.3	17	1.4
Model 2 (with BW22 as covariate)				
BrW	1	114–115	349	1.54
	18	1.0–2.0	632	1.02
	33	3.1–3.2	17	4.07
DW	1	194–195	359	1
	7	34–35	451	2.09
	15	8.0–9.0	608	1.34
TW	1	166–167	356	1.08
WW	1	111–112	363	1.02
	2	129–130	254	3.13
	4	75–76	263	4.92
	Z	14–15	284	1.14
BaW	4	68–69	349	2
HW	3	17–18	379	3.44
	10	3.0–4.0	579	1.01
NeW	1	12–13	348	1.16
	1	147–148	418	1.22
	1	170–171	345	7.75
	4	75–76	263	7.57
	9	2.0–3.0	524	1.75
ShW	1	164–165	346	1.21
	1	170–171	345	11.27
	4	75–76	263	36.61
GzW	1	170–171	345	6.84
	4	22–23	288	1.25
	4	75–76	263	12.86
LiW	4	75–76	263	24.43
HeW	1	170–171	345	2.52
	4	75–76	263	2.54
	6	30–31	444	1.17
	7	18–19	440	1.92
	33	3.2–3.3	17	1.29

BW22, Body weight at week 22; ADG, Average daily gain; DrW, Dressed weight; BrW, Breast weight; DW, Drumstick weight; TW, Thigh weight; WW, Wing weight; BaW, Back weight; HW, Head weight; NeW, Neck weight; ShW, Shank weight; GzW, Gizzard weight; LiW, Liver weight; HeW, Heart weight; Chr, Chromosome.

**TABLE 6:** Positions and genes located in 1-Mb windows that explained ≥ 1% of genetic variance for growth, carcass, and internal organ traits (Model 1: without BW22 as a covariate).

Trait	Chr	1-Mb window	Genes
BW 22	1	169–170	*LSAMP, EPHAS, CADM2, ROBO1, ROBO2, DSCAM, DMD, GPC5, GPC6, PCDH9, NBEA, CNTNS, FAT3, DLG2, TENM4, IL1RAPL1, FAM155A*
	1	170–171	*KPNA3, CAB39L, CDADC1, RCBTB1, RCBTB2, ARL11, SPRYD7, TRIM13, KCNRG, MIR16-1, MIR15-A, SETDB2, MLNR, CRSLTR2, LPAR6, RB1, ITM2B, FNDC3A, MED4*
	1	171–172	*RNASEH2B, INTS6, FAM124A, SERPINE3, DLEU7, WDFY2, DHRS12, TMEM272, ATP7B, ALG11, NEK3, NEK5, CKAP2, VPS36, THSD1, FGL1L, TPTE2, SLC25A15, MRPS31, FOXO1*
	2	22–23	*CDK6, CDK14, FZD1, AKAP9, CYP51A1, KRIT1, NK1B1, GATAD1, ACCSL, PEX1, RBM48, EFCAB1, FAM133B, MIR1650, SAMD9L, HEPACAM2, VPS50*
	2	39–40	*RBMS3, TGFBR2, GADL1*
	2	110–111	*XKR4, RGS20, TCEA1, LYPLA1, MRPL15, SOX17, RP1, RB1CC1, NPBWR1, ATPV61H, QPRK1*
	4	76	*SLIT2, LCORL, FAM184B, NCARPG, QDPR, LAP3, MED28, MIR218-1*
	5	27–28	*SMOC1, COX16, SLC8A3, SIPA1L1, RGS6, PCNX1, MAP3K9, TTC9, MED6, SYNJ2BP*
ADG	1	133–134	*ATP10A, UBE3A, CNGA3, VWA3B, COA5, UNC50, MGAT4A, KIAA1211L, TSGA10, LIPT1, MITD1, MRPL30, LYGL, LYGL2, TXNDC9, EIF 5B, REV1, AFF3*
	1	170–171	*KPNA3, CAB39L, CDADC1, RCBTB1, RCBTB2, ARL11, SPRYD7, TRIM13, KCNRG, MIR16-1, MIR15-A, SETDB2, MLNR, CRSLTR2, LPAR6, RB1, ITM2B, FNDC3A, MED4*
	1	171–172	*RNASEH2B, INTS6, FAM124A, SERPINE3, DLEU7, WDFY2, DHRS12, TMEM272, ATP7B, ALG11, NEK3, NEK5, CKAP2, VPS36, THSD1, FGL1L, TPTE2, SLC25A15, MRPS31, FOXO1*
	4	75–76	*SLIT2, LCORL, FAM184B, NCARPG, QDPR, LAP3, MED28, MIR218-1*
	33	3.2–3.3	*SCN8A, FIGNL2, ANKRD33, ACVRL1, ACVR1B*
DrW	1	170–171	*KPNA3, CAB39L, CDADC1, RCBTB1, RCBTB2, ARL11, SPRYD7, TRIM13, KCNRG, MIR16-1, MIR15-A, SETDB2, MLNR, CRSLTR2, LPAR6, RB1, ITM2B, FNDC3A, MED4*
	1	171–172	*RNASEH2B, INTS6, FAM124A, SERPINE3, DLEU7, WDFY 2, DHRS12, TMEM272, ATP7B, ALG11, NEK3, NEK5, CKAP2, VPS36, THSD1, FGL1L, TPTE2, SLC25A15, MRPS31, FOXO1*
	4	75–76	*SLIT2, LCORL, FAM184B, NCARPG, QDPR, LAP3, MED28, MIR218-1*
	18	7.0–8.0	*CEP112, NOL11, PITPNC1, PSMD12, HELZ, CACNG1, CACNG4, CACNG5, PRKCA, TRNAR-CCG, APOH, AXIN2, RGS9, GNA13, ARSG, SLC16A6, WIPI1, PRKAR1A, FAM20, ABCA5, ABCA8, ABCA9, MAP2K6*
BrW	1	18.0–19.0	*BRD1, ZBED4*
	1	170–171	*KPNA3, CAB39L, CDADC1, RCBTB1, RCBTB2, ARL11, SPRYD7, TRIM13, KCNRG, MIR16-1, MIR15-A, SETDB2, MLNR, CRSLTR2, LPAR6, RB1, ITM2B, FNDC3A, MED4*
	1	171–172	*RNASEH2B, INTS6, FAM124A, SERPINE3, DLEU7, WDFY 2, DHRS12, TMEM272, ATP7B, ALG11, NEK3, NEK5, CKAP2, VPS36, THSD1, FGL1L, TPTE2, SLC25A15, MRPS31, FOXO1*
	1	180–181	*ZMYM2, LATS2, XPO4, EEF1AKMT1, IL17D, IFT88, CRYL1, GJB2, GJA3, PSPC1, MIR6641, MPHOSPH8, PARP4, CENPJ, RNF17, ARHGAP20, FDX1, RDX, ZC3H12C*
	1	182–183	*CWF19L2, VMO1, GUCY1A2, MIR1709, AASDHPPT, KBTBD3, MSANTD4, GRIA4*
	4	75–76	*SLIT2, LCORL, FAM184B, NCARPG, QDPR, LAP3, MED28, MIR218-1*
	33	3.2–3.3	*SCN8A, FIGNL2, ANKRD33, ACVRL1, ACVR1B*
DW	1	170–171	*KPNA3, CAB39L, CDADC1, RCBTB1, RCBTB2, ARL11, SPRYD7, TRIM13, KCNRG, MIR16-1, MIR15-A, SETDB2, MLNR, CRSLTR2, LPAR6, RB1, ITM2B, FNDC3A, MED4*
	4	75–76	*SLIT2, LCORL, FAM184B, NCARPG, QDPR, LAP3, MED28, MIR218-1*
TW	1	169–170	*LSAMP, EPHAS, CADM2, ROBO1, ROBO2, DSCAM, DMD, GPC5, GPC6, PCDH9, NBEA, CNTNS, FAT3, DLG2, TENM4, IL1RAPL1, FAM155A*
	1	170–171	*KPNA3, CAB39L, CDADC1, RCBTB1, RCBTB2, ARL11, SPRYD7, TRIM13, KCNRG, MIR16-1, MIR15-A, SETDB2, MLNR, CRSLTR2, LPAR6, RB1, ITM2B, FNDC3A, MED4*
	1	171–172	*RNASEH2B, INTS6, FAM124A, SERPINE3, DLEU7, WDFY 2, DHRS12, TMEM272, ATP7B, ALG11, NEK3, NEK5, CKAP2, VPS36, THSD1, FGL1L, TPTE2, SLC25A15, MRPS31, FOXO1*
	4	75–76	*SLIT2, LCORL, FAM184B, NCARPG, QDPR, LAP3, MED28, MIR218-1*
WW	1	170–171	*KPNA3, CAB39L, CDADC1, RCBTB1, RCBTB2, ARL11, SPRYD7, TRIM13, KCNRG, MIR16-1, MIR15-A, SETDB2, MLNR, CRSLTR2, LPAR6, RB1, ITM2B, FNDC3A, MED4*
	1	171–172	*RNASEH2B, INTS6, FAM124A, SERPINE3, DLEU7, WDFY 2, DHRS12, TMEM272, ATP7B, ALG11, NEK3, NEK5, CKAP2, VPS36, THSD1, FGL1L, TPTE2, SLC25A15, MRPS31, FOXO1*
	1	195–196	*UVRAG, LRRC32, GUCY2F, ENSY, THAP12, TRNAP-AGG, TRNAP-UGG, WNT11, ART1, ART7B, ART7C, MADPRT1, IL18BP, RNF121, RNF169, TRPC2L, NUMA1, LAMTOR1, LRTOMT, ANAPC15, WDR73, ADAM15, SLCO2B1, TPBGL, PGM2L1, KCNE3, LIPT2, POLD3, CHRDL2, XRRA1*
	4	75–76	*SLIT2, LCORL, FAM184B, NCARPG, QDPR, LAP3, MED28, MIR218-1*
BaW	1	171–172	*RNASEH2B, INTS6, FAM124A, SERPINE3, DLEU7, WDFY 2, DHRS12, TMEM272, ATP7B, ALG11, NEK3, NEK5, CKAP2, VPS36, THSD1, FGL1L, TPTE2, SLC25A15, MRPS31, FOXO1*
	4	66–67	*SGCB, SPATA18, OCIAD1, LRRC66, DCUN1D4, CWH43, FRYL, CORIN, GABRA4, TEC, SLAIN2, CNGA1, NFXL1, NIPAL1, TXK, ZAR1, SLC10A4, ATP10D, COMMD8*
	4	75–76	*SLIT2, LCORL, FAM184B, NCARPG, QDPR, LAP3, MED28, MIR218-1*
HW	1	170–171	*KPNA3, CAB39L, CDADC1, RCBTB1, RCBTB2, ARL11, SPRYD7, TRIM13, KCNRG, MIR16-1, MIR15-A, SETDB2, MLNR, CRSLTR2, LPAR6, RB1, ITM2B, FNDC3A, MED4*
	4	75–76	*SLIT2, LCORL, FAM184B, NCARPG, QDPR, LAP3, MED28, MIR218-1*
	8	27–28	*CYP2J 19, CYP2J24P, CYP2J21, CYP2J22, CYP2J23, NFIA, TM2D1, PATJ, USP1, KANK4, ANGPTL3, DOCK7*
NeW	1	12–13	*MAGI2, TMEM60, PTPN12, PHTF2, RSBN1L, GSAP, LRRC17, CCDC146, FAM185A, FGL2*
	1	170–171	*KPNA3, CAB39L, CDADC1, RCBTB1, RCBTB2, ARL11, SPRYD7, TRIM13, KCNRG, MIR16-1, MIR15-A, SETDB2, MLNR, CRSLTR2, LPAR6, RB1, ITM2B, FNDC3A, MED4*
	4	75–76	*SLIT2, LCORL, FAM184B, NCARPG, QDPR, LAP3, MED28, MIR218-1*
	9	2–3	*ARHGEF4, PLEKHB2, FAM168B, CLDN15, PARL, AMER3, MAP6D1, YEATS2, DUSP28, GPC1, KLHL6, KLHL24, GPR148*
ShW	1	164–165	*PCDH17*
	1	170–171	*KPNA3, CAB39L, CDADC1, RCBTB1, RCBTB2, ARL11, SPRYD7, TRIM13, KCNRG, MIR16-1, MIR15-A, SETDB2, MLNR, CRSLTR2, LPAR6, RB1, ITM2B, FNDC3A, MED4*
	4	75–76	*SLIT2, LCORL, FAM184B, NCARPG, QDPR, LAP3, MED28, MIR218-1*
GzW	1	170–171	*KPNA3, CAB39L, CDADC1, RCBTB1, RCBTB2, ARL11, SPRYD7, TRIM13, KCNRG, MIR16-1, MIR15-A, SETDB2, MLNR, CRSLTR2, LPAR6, RB1, ITM2B, FNDC3A, MED4*
	4	22–23	*RAPGEF2, C4H4ORF45, FSTL5*
	4	75–76	*SLIT2, LCORL, FAM184B, NCARPG, QDPR, LAP3, MED28, MIR218-1*
LiW	4	75–76	*SLIT2, LCORL, FAM184B, NCARPG, QDPR, LAP3, MED28, MIR218-1*
HeW	1	155–156	*SLAIN1, EDNRB, SCEL, MYCBP2, FBXL3, ES1ML1, ACOD1, KCTD12, CLN5*
	1	170–171	*KPNA3, CAB39L, CDADC1, RCBTB1, RCBTB2, ARL11, SPRYD7, TRIM13, KCNRG, MIR16-1, MIR15-A, SETDB2, MLNR, CRSLTR2, LPAR6, RB1, ITM2B, FNDC3A, MED4*
	4	75–76	*SLIT2, LCORL, FAM184B, NCARPG, QDPR, LAP3, MED28, MIR218-1*
	6	30–31	*HTN1, SLC18A2, VAX1, KCNK18, PDZD8, EMX2, RAB11FIP2, FAM204A, CACUL1, PRLHR, GRK5, EIF3A, FAM45A, NANOS1, PRDX3, SFXN4*
	7	18–19	*TLK1, DCAF17, CYBRD1, GAD1, GORASP2, SP5, MYO3B, CCDC173L, METTL5, SSB, UBR3, KLHL23, PHOSPHO2, KLHL41, FASTKD1, PPIG, BBS5, LRP2, ABCB11, G6PC2, RDH7L, SPC25, MIR1733*
	13	6–7	*TENM2*
	33	3.2–3.3	*SC8A, FIGNL2, ANKRD33, ACVR1B, ACVRL1*

BW22, Body weight at week 22; ADG, Average daily gain; DrW, Dressed weight; BrW, Breast weight; DW, Drumstick weight; TW, Thigh weight; WW, Wing weight; BaW, Back weight; HW, Head weight; NeW, Neck weight; ShW, Shank weight; GzW, Gizzard weight; LiW, Liver weight; HeW, Heart weight; Chr, Chromosome.

Italic represent Names of Genes.

**TABLE 7:** Positions and genes located in 1-Mb windows which explain ≥1% of genetic variance for growth, carcass, and internal organ traits (Model 2, with BW22 as a covariate).

TRAIT	Chr	1 Mb window	Genes
BrW	1	114–115	*TSPAN7, MID1TP1, OTP, RPGR, SRPX, SYTL5, DYNLT3, CYBB, XK, LANCL3, PRRG1, C1HXORF59*
	18	1.0–2.0	*MYH10, ELAC2, DNAH9, TRNAM-CAU, PIRT, SHISA A6, RNF222, NDEL1, CCDC42, PIK3R5, PIK3R6*
	33	3.2–3.3	*SCN8A, FIGNL2, ANKRD33, ACVRL1, ACVR1B*
DW	1	194–195	*USP35, KCTD14, KCTD21, ALG8, NDUFC2, THRSP, THRSPB, INTS4, AAMDC, RSF1, CLN1A, AQP11*
	7	34–35	*MBD5, ACVR2A, ORC4, EPC2, KIF5C, LYPD6, LYPD6B, MMADHC, MIR1C*
	15	8.0–9.0	*BCR, SMARCB1, DERL3, SLC2A11, SLC2A11L1, SLC2A11L2, SLC2A11L3, SLC2A11L4, MIF, DDX51, GSTT1L, DDT, CABIN1, CRKL, KLHL22, MED15, SMPD4, GGT5, GTT1, GGT2, LRRC75B, SNRPD3, GUCD1, UPB1, ADORA2A, SPECC1L, RAB36, RSPH14, GNAZ, ZNRF3, XBP1, KREMEN1, SUSD2, SBSPONL, GSC2, DGCR2, CA15L, IGLL1, VPS29L, VPREB3, CHCHD10, MMP11, TBX6*
TW	1	166–167	*OLFM4*
WW	1	111–112	*CBS, U2AF1, CRYAA, SIK1, HSF2BF, RRP1B, PDXK, AGPAT3, TRAPPC10, PWP2, C1H21ORF33, VTCN1L, ICOSLG, MIR221, MIR222*
	2	129–130	*RIMS2, SLC25A32, DCSTAMP, DPYS, LRP12, UBR5, ODF1, KLF10, AZIN1, ATP6V1C1, BAALC, FZD6, CTHRC1*
	4	75–76	*SLIT2, LCORL, FAM184B, NCARPG, QDPR, LAP3, MED28, MIR218-1*
	Z	14–15	*HCN1, MRPS30, FGF10, EMB, PARP8*
BaW	4	68–69	*ATP8A1, GRXCR1, SLC30A9, BEND4, SHISA3, PHOX2B, TMEM33, APBB2, UCHL1, LIMCH1, RBM47, NSUN7, CHRNA9*
HW	3	17–18	*LIN9, C3H1orf95, PARP1, TRMT6, CRLS1, ACBD3, MIXL1, TEM63A, SDE2, ENAH, H3F3C, LEFTY2, SRP9, EPHX1, LBR, DNAH14, CNIH3, CNIH4, WDR26, NVL, TP53BP2, FBXO28, CAPN2, CAPN8, DEGS1, TLR5, SUSD4*
	10	30–40	*NEIL1, ZP3, ZP3L1, ISLR.ISLR2, ISL2, PML, PMLL, CCDC33, COMMD4, PTPN9, STOML1, CYP11A1, SIN3A, LINGO1, CSPG4, MAN2C1, SNX33, SNUPN, HMG20A, PEAK1, TSPAN3, SCAPER, RCN2, PSTPIP1, ETFA, TMEM266, NRG4, FBXO22, UBE2Q2*
NeW	1	12–13	*MAGI2, TMEM60, PTPN12, PHTF2, RSBN1L, GSAP, LRRC17, CCDC146, FAM185A, FGL2*
	1	147–148	*HS6ST3, UGGT2, DZIP1, DNAJC3, TRNAF-GAA, ABCC4, CLDN10, GPR180, DCT, TGDS, SOX21*
	1	170–171	*KPNA3, CAB39L, CDADC1, RCBTB1, RCBTB2, ARL11, SPRYD7, TRIM13, KCNRG, MIR16-1, MIR15-A, SETDB2, MLNR, CRSLTR2, LPAR6, RB1, ITM2B, FNDC3A, MED4*
	4	75–76	*SLIT2, LCORL, FAM184B, NCARPG, QDPR, LAP3, MED28, MIR218-1*
	9	2–3	*ARHGEF4, PLEKHB2, FAM168B, CLDN15, PARL, AMER3, MAP6D1, YEATS2, DUSP28, GPC1, KLHL6, KLHL24, GPR148*
ShW	1	164–165	*PCDH17*
	1	170–171	*KPNA3, CAB39L, CDADC1, RCBTB1, RCBTB2, ARL11, SPRYD7, TRIM13, KCNRG, MIR16-1, MIR15-A, SETDB2, MLNR, CRSLTR2, LPAR6, RB1, ITM2B, FNDC3A, MED4*
	4	75–76	*SLIT2, LCORL, FAM184B, NCARPG, QDPR, LAP3, MED28, MIR218-1*
GzW	1	170–171	*KPNA3, CAB39L, CDADC1, RCBTB1, RCBTB2, ARL11, SPRYD7, TRIM13, KCNRG, MIR16-1, MIR15-A, SETDB2, MLNR, CRSLTR2, LPAR6, RB1, ITM2B, FNDC3A, MED4*
	4	22–23	*RAPGEF2, C4H4ORF45, FSTL5*
	4	75–76	*SLIT2, LCORL, FAM184B, NCARPG, QDPR, LAP3, MED28, MIR218-1*
LiW	4	75–76	*SLIT2, LCORL, FAM184B, NCARPG, QDPR, LAP3, MED28, MIR218-1*
HeW	1	170–171	*KPNA3, CAB39L, CDADC1, RCBTB1, RCBTB2, ARL11, SPRYD7, TRIM13, KCNRG, MIR16-1, MIR15-A, SETDB2, MLNR, CRSLTR2, LPAR6, RB1, ITM2B, FNDC3A, MED4*
	4	75–76	*SLIT2, LCORL, FAM184B, NCARPG, QDPR, LAP3, MED28, MIR218-1*
	6	30–31	*HTN1, SLC18A2, VAX1, KCNK18, PDZD8, EMX2, RAB11FIP2, FAM204A, CACUL1, PRLHR, GRK5, EIF3A, FAM45A, NANOS1, PRDX3, SFXN4*
	7	18–19	*TLK1, DCAF17, CYBRD1, GAD1, GORASP2, SP5, MYO3B, CCDC173L, METTL5, SSB, UBR3, KLHL23, PHOSPHO2, KLHL41, FASTKD1, PPIG, BBS5, LRP2, ABCB11, G6PC2, RDH7L, SPC25, MIR1733*
	33	3.2–3.3	*SC8A, FIGNL2, ANKRD33, ACVR1B, ACVRL1*

BrW, breast weight; DW, drumstick weight; TW, thigh weight; WW, Wing weight; BaW, Back weight; HW, Head weight; NeW, neck weight; ShW, Shank weight; GzW, Gizzard weight; LiW, Liver weight; HeW, Heart weight; Chr, Chromosome.

Italic represent Names of Genes.

Eight 1-Mb windows of SNPs that explained more than 1% of the genetic variance of body weight at week 22 were found. Two of these windows on chromosomes 1 and 4 explained more than 10% of the genetic variance of this trait (see Supplementary Figure S1A). These regions contained several annotated genes including *SLIT2, LCORL, NCARPG, LAP3, MED28, INTS6, DLEU7, CKAP2, KPNA3,* and *CAB39L* (Table 6).

Five 1-Mb windows of SNPs explained more than 1% of the genetic variance of ADG (Supplementary Figure S1B). One of these windows located on chromosome 4 explained more than 10% of the genetic variance of ADG. This window contained several genes including *SLIT2, LCORL, FAM184B, NCARPG, QDPR, LAP3, MED28,* and *MIR218-1* (Table 6).

For breast weight, seven 1-Mb SNP windows on chromosomes 1, 4, and 33 reached the level of significance (see Supplementary Figure S2C). These windows together explained about 20.5% of the genetic variance of this trait. Many genes including *RNASEH2B, INTS6, SERPINE3, DLEU7, WDFY2, NEK3, NEK5, CKAP2, TPTE 2, SLC25A15, FOXO1, SLIT2, LCORL, NCARPG, LAP3, SCN8A, FIGNL2, ANKRD33, ACVRL1,* and *ACVR1B* were found in these regions (Table 6). With BW22 included as a covariate (Model 2), the number of significant SNP windows decreased from seven to three and explained only about 6.63% of the genetic variance of this trait. Within these SNP windows, several genes, including *SCN8A, FIGNL2, ANKRD33, ACVRL1, ACVR1B, MYH10, ELAC2, DNAH9, TRNAM-CAU, PIRT, SHISA A6, RNF222, NDEL1, CCDC42, PIK3R5, PIK3R6* were co-located on chromosome 1, chromosome 18 and chromosome 33 (Table 7).

Two SNP windows on chromosomes 1 and 4 explained 32.5% of the genetic variance of drumstick weight (Supplementary Figure S2D). These windows contained 34 annotated genes *including SLIT2, LCORL, NCARPG, LAP3, MED28, CLRN2, MIR218-1, KPNA3, CAB39L, CDADC1, RCBTB1, ARL11, SPRYD7, TRIM13, KCNRG, MIR15-A, SETDB2, LPAR6,* and *MED4* (Table 6). However, with BW22 as a covariate, the number of significant SNP windows increased from two to three, but they explained only 4.43% of the genetic variance of this trait. Seventy-two genes on chromosomes 1, 7, and 15 were observed. These include *MBD5, ACVR2A, ORC4, EPC2, KIF5C, LYPD6, MMADHC, MIR1C, DERL3, SLC2A11, MYO7A and CAPN5* (Table 7).

One SNP window on chromosome 4 and three SNP windows on chromosome 1 explained about 40.9% of the genetic variance of WW (Supplementary Figure S3E). These regions contained many genes some of which are *UVRAG, LRRC32, GUCY2F, ENSY, THAP12, TRNAP-AGG, TRNAP-UGG, WNT11, ART1, ART7B, ART7C, MADPRT1, IL18BP, RNF121, RNF169, TRPC2L, NUMA1, LAMTOR1, LRTOMT, ANAPC15, WDR73, ADAM15, SLCO2B1, TPBGL, PGM2L1, KCNE3, LIPT2, POLD3, CHRDL2,* and *XRRA1* (Table 6). With BW22 as a covariate, four SNP windows on chromosomes 1, 2, 4 and Z were significant, and together they explained 10.21% of the genetic variance of WW (Table 7).

Four SNP windows on chromosomes 1 and 4 explained 31.8% of the genetic variance of thigh weight (Supplementary Figure S3F). Many annotated genes, including *LSAMP, EPHAS, CADM2, ROBO1, DSCAM, DMD, GPC5, PCDH9, NBEA, CNTNS, FAT3, DLG2, TENM4, IL1RAPL1, FAM155A, RNASEH2B, INTS6, FAM124A, SERPINE3, DLEU7, WDFY2, DHRS12, TMEM272, ATP7B, ALG11, NEK3, NEK5, CKAP2, VPS36, THSD1, FGL1L, TPTE2, SLC25A15, MRPS31,* and *FOXO1* were observed within these regions (Table 6). With the inclusion of BW22 as a covariate, only 1 SNP window on chromosome 1 was significant and explained about 1.01% of the genetic variances for thigh weight. This genomic region contained *OLFM4* (Table 7).

One SNP window on chromosome 4 explained about 14% of the genetic variance of dressed weight (Supplementary Figure S4G). This region contained *SLIT2, LCORL, FAM184B, NCARPG, QDPR, LAP3*, *MED28, MIR218* (Table 6).

Three SNP windows, two on chromosome 4 and one on chromosome 1, explained about 11% of the genetic variance of back weight (Supplementary Figure S4H). These genomic regions contained many genes as shown in Table 6. With BW22 as a covariate, only 2% of the genetic variance was explained by one SNP window on chromosome 4.

A total of three SNP windows on chromosomes 1, 4, and 8 explained about 5.5% of the genetic variance of head weight (Supplementary Figure S5I). The genes in these genomic regions can be seen in Table 6. The inclusion of BW22 as a covariate had two SNP windows on chromosomes 3 and 10 explaining 4.45% of the genetic variance of this trait and the genes in these genomic regions are shown in Table 7.

About 14.6% of the genetic variance of neck weight was explained by four SNP windows. Two of these SNPs were on chromosome 1 while the rest were on chromosomes 4 and 9 (Supplementary Figure S5J). The inclusion of BW22 as a covariate increased the amount of genetic variance explained by the same SNP windows to 19.45%. The annotated genes located in these genomic regions are shown in Tables 6, 7.

Two SNP windows on chromosome 1 and one SNP window on chromosome 4 explained about 44.5% of the genetic variance of shank weight (Supplementary Figure S6K). The inclusion of BW22 as a covariate increased the amount of genetic variance explained by the same SNP windows to 49.1%. Tables 6, 7 show the annotated genes that are found in these genomic regions.

Two SNP windows on chromosome 4 and one SNP window on chromosome 1 explained about 23.7% of the genetic variance of gizzard weight (Supplementary Figure S6L). The inclusion of BW22 as a covariate decreased the amount of genetic variance explained by the same SNP windows to 20.95%. Tables 6, 7 show the annotated genes that are found in these genomic regions.

One SNP window on chromosome 4 explained 20.5% of the genetic variance of liver weight (Supplementary Figure S7M). The inclusion of BW22 as a covariate increased the amount of genetic variance explained by this SNP windows to 24.43%. This window contained several genes (see Tables 6, 7).

About 12% of the genetic variance of heart weight was explained by seven SNP windows. Two of these were on chromosome 1 while the rest were on chromosomes 4, 6, 7, 13, and 33 (Supplementary Figure S7N). With the inclusion of BW22 as a covariate, the above-mentioned SNP windows, except for the SNP window on chromosome 13, explained 9.44% of the genetic variance of this trait. The annotated genes located in these genomic regions can also be seen in Tables 6, 7.

## 4 Discussion

### 4.1 Population structure

The admixture analysis based on identity by state (Figure 1), indicates that notwithstanding the evidence of admixture, all the three chicken ecotypes of Ghana appeared to have come from three distinct ancestral populations. Similar observations have been reported by Osei-Amponsah et al. (2010b). In another study involving the same chicken ecotypes, some of which were related to those used in this study, Walugembe et al. (2020) also arrived at the same conclusion. The IS ecotype appeared distinct from the CS and FO ecotypes, which, on the other hand, appeared to have a somewhat similar ancestry. The admixture of the CS and FO ecotypes could be a result of significant gene flows between the forest and coastal agroecological zones due to their proximity to each other.

### 4.2 Genetic parameters

Estimates of heritabilities for the growth and carcass traits of the three chicken ecotypes of Ghana, without BW22 as a covariate, as shown in Tables 1, 3 ranged from a low of 0.17 for BaW to a high of 0.69 for ADG. These estimates generally agree with the findings of several authors, including Rance et al. (2002), Venturini et al. (2014), El-Attroun et al. (2017), and El-Attroun et al. (2021). On the other hand, estimates of heritability for the internal organs ranged from a low of 0.13 for LiW to a medium of 0.35 for GzW. Heritability estimates for HeW and GzW are similar to those reported by Gaya et al. (2006), Venturini et al. (2014), and Dou et al. (2019) while the estimate of heritability for LiW was also similar to the findings of Moriera et al. (2019) but different from those of Venturini et al. (2014) and Dou et al. (2019). Growth traits of unselected chicken populations tend to have relatively high heritability. For example, Walugembe et al. (2020) also found a heritability for growth rate even after a challenge with La Sota Newcastle Disease Virus strain of above 0.4, and a pre-challenge heritability of 0.55.

Inclusion of BW22 as a covariate did not significantly affect heritability estimates of any of the internal organ traits (Table 4). However, with BW22 as a covariate, estimates of heritabilities of the carcass traits (Table 2) ranged from 0.21 for TW to 0.38 for WW. Direct selection could therefore be effective in improving some of these traits in Ghanaian local chicken populations.

High positive genetic and phenotypic correlations were found between most of the traits, except between DP and BW22 and between DP and ADG, which had low genetic and phenotypic correlation estimates. HW also had low phenotypic correlations with GzW and LiW. Estimates of the genetic correlation of BW22 with ADG, DrW, BrW, TW, WW, and DW were positive and high, suggesting that these traits could make indirect genetic gains when selection is directed at increasing BW22. Furthermore, the estimate of the genetic correlation between LiW and HeW was also high and similar to the findings of Rance et al. (2002) and Gaya et al. (2006). Inclusion of BW22 as covariate did not change the magnitude and direction of estimates of the phenotypic and genetic correlations for most carcass traits but led to a reduction in correlation estimates between the internal organ traits. Moderate to strong positive genetic and phenotypic correlations of body weight with carcass traits of chicken have also been reported by Venturini et al. (2014), Bungsrisawat et al. (2018), and El-Attrouny et al. (2021) but these conclusions are at variance with those of Osei-Amponsah (2010a), who reported weak to moderate negative phenotypic correlations between live weight and most carcass traits of Forest and Savannah chicken populations of Ghana. The small differences between estimates of the phenotypic *versus* the genetic correlations suggest that the environmental correlation was of similar magnitude as the genetic correlation. Furthermore, the medium to strong positive genetic correlation estimates between some of the growth, carcass, and internal organ traits of local chicken ecotype populations in Ghana suggests that selection based on body weight could enhance some of the carcass traits.

### 4.3 Positional candidate genes for growth traits of local chicken

Body weight is a polygenic trait, and chromosomes 1 and 4 of the chicken genome have been widely reported to harbour QTL for growth (Podisi, et al., 2013; Mebratie et al., 2019; Wang et al., 2022). In this study, a 1-Mb SNP window on chromosome 4 explained 19.7% and 11.6% of the genetic variance for BW22 and ADG, respectively, while another SNP window on chromosome 1 also explained 10.4% and 9.6% of the genetic variance of BW22 and ADG. These two chromosomal regions contain many genes, some of which have previously been reported to be associated with growth and carcass traits in chicken and other farm animals (Yang et al., 2021; Wang et al., 2022). The genes in these genomic regions include ligand dependent nuclear receptor corepressor like (*LCORL*) and non-SMC condensin I complex subunit G (*NCAPG*), which play an important role in arginine metabolism and are linked with growth in animals (Wu et al., 2009; Tetens et al., 2013; Tiensuu et al., 2019); leucine aminopeptidase 3 (*LAP3*) and LIM domain binding 2 (*LDB2*) genes which have an influence on growth traits of chicken (Gu et al., 2011). SNPs in karyopherin subunit alpha 3 (*KPNA3*) and *RCBTB1* genes are also associated with growth in chicken (Wang et al., 2022; Zhu et al., 2023). Calcium binding protein 39 like (*CAB39L*) which is on chromosome 1 plays an important role in the regulation of food intake by activating AMP-activated protein kinase through the process of phosphorylation (Proszkowiec et al., 2006) and regulates body weight in chicken (Li et al., 2021; Zhang et al., 2021; Zhu et al., 2023). Some SNPs in the deleted lymphocytic leukemia 7 (*DLEU7*) gene have also been reported by Abdalhag et al. (2015) to be associated with growth traits in Jinghai yellow chickens. Forkhead box O1 (*FOXO1*) is another gene that has also been widely reported to influence average daily intake and the formation of adipose tissue and skeletal muscle of chickens (Xie et al., 2012). Xie et al. (2012) also observed that some SNPs in *INTS6* are significantly associated with body weight of chicken at 90 days of age.

### 4.4 Positional candidate genes for carcass traits of local chicken

Carcass traits in chickens are also influenced by many genes with small individual effects. Genes associated with breast muscle weight, drumstick weight, thigh weight, wing weight, dressed weight, head weight, back weight, neck weight, and shank weight were mainly located on chromosomes 1 and 4. Some of these genes have been reported in the literature, including *FOXO1,* which plays an important role in muscle development by mediating PI3K-AKT-MAPK and PI3K-AKT-mTOR pathways (Xie et al., 2012; Jia et al., 2017). *LCORL,* a gene on chromosome 4, is reported to be expressed at higher levels in the breast muscle of high-muscle-weight chickens than in low-muscle-weight chickens (Liu et al., 2015). *SLIT2* plays a regulatory role in the differentiation of osteoblast (Sun et al., 2009) and the inhibition of bone resorption (Park et al., 2019) and *KPNA3* influences growth and muscle quality in chicken (Pértille et al., 2015; Li et al., 2022). *LAP3* and *FAM184B* have been associated with organ weight in cattle and sheep (An et al., 2018; La et al., 2019). *SERPINE3*, one of the serine proteinase inhibitor (serpin) gene family members, and *INTS6* are associated with bone quality (Guo et al., 2017) while Mediator Complex Subunit 4 (*MED4*) regulating vitamin D metabolism also affects development and maintenance of mineral ion homeostasis and skeletal integrity (Sutton and MacDonald, 2003). *MLNR* gene encodes a motilin receptor that promotes the release of growth hormone. In chicken, motilin receptor is largely involved in gastrointestinal functions including increments in Ca^+2^ levels and is associated with bone traits (Takahashi et al., 2014; Li et al., 2021). *FNDC3A* is also associated with bone traits (Li et al., 2021).

### 4.5 Positional candidate genes for internal organ traits of local chicken

The internal organs of animals are highly nutritious and contain high levels of bioavailable protein, amino acids, vitamins, and micronutrients (Fayemi et al., 2018). They are relatively cheaper than other meats and are easily available. As a result, their consumption among low-income and food insecure households in developing countries is quite high. Selective breeding aimed at improving the internal organ traits of chicken could therefore play a significant role in improving nutritional outcomes amongst children and low income-households in developing countries.

Chromosomes 1 and 4 contained several genes that exhibited a pleiotropic effect in gizzard, liver, and heart weights. These include *SLIT2, LCORL, NCARPG, QDPR, LAP3, MED28, KPNA3, CAB39L, SPRYD7, TRIM13, KCNRG, SETDB2, MLNR, CRSLTR2, LPAR6, RB1, ITM2B, FNDC3A,* and *MED4.* Other significant genes that are associated with internal organ traits of chicken include Empty spiracles homeobox 2 (*EMX2*) which is on chromosome 6 and is associated with heart weight. This gene plays a major role in transcriptional regulation of the slow myosin heavy chain 2 (MyHC2) gene in fast/slow embryonic muscle fibers (Weimer et al., 2013). Other genes that were found to be associated with heart weight include: *CDK2* Associated Cullin Domain (*CALCUL1*) which is on chromosome 6 and is implicated in positive regulation of cell population proliferation and protein kinase activity (Kong et al., 2009; Zhang et al., 2021); Activin A receptor type 1B (*ACVR1B*) gene which encodes an activin A type IB receptor. Activins are dimeric growth and differentiation factors which belong to the transforming growth factor-beta (TGF-beta). SNPs in (TGF)-β2, 3, and 4 have been reported by Hosnedlova et al. (2020) to be associated with growth, skeletal and body composition traits of chicken; SLAIN motif family member 2 (*SLAIN2*) is involved in cytoplasmic microtubule organization (van der Vaart et al., 2011); Retinoblastoma 1 (*RB1*) is associated with body weight and bone traits in chicken (Zhang et al., 2011); Motilin receptor (*MLNR*) gene which encodes a motilin receptor and is also associated with growth and bone traits in chicken (Takahashi et al., 2014). Some significant positional genes for gizzard weight include the Follistatin like 5 (*FSTL5*) which is predicted to facilitate calcium ion binding activity and cell differentiation (Zhang et al., 2017); Calcium voltage-gated channel auxiliary subunit beta 1 (*CACNB1*) which affects skeletal muscle development in mice (Chen et al., 2011); *SLAIN2; RB1*; *MLNR*; *SAMD9* and *FNDC3A*. A SNP window on chromosome 4 which explained about 21% of the genetic variance of liver weight contained *SLIT2, LCORL, FAM184B, NCARPG, QDPR, LAP3, MED28, MIR218-1* genes.

## 5 Conclusion

We estimated genetic parameters and performed GWAS for several growth, carcass, and internal organ traits in local Ghanaian chicken ecotypes. The results show that heritabilities for growth and carcass traits were moderate to high, while the genetic correlations between these traits were generally positively high. The moderate to high heritabilities of BW22, ADG, dressed weight, drumstick weight, thigh weight, breast weight, wing weight, head weight, neck weight, shank weight, and gizzard weight indicates that these traits could be improved in these populations through selective breeding.

A total of 58 1-Mb SNP windows each of which explained more than 1% of the genetic variance of the growth, carcass, and internal organ traits studied contained many genes including *EMX2, CALCUL1, ACVR1B, CACNB1, RB1, MLNR, FOXO1, NCARPG, LCORL, LAP3, LDB2*, *KPNA3, DLEU7* and *CAB39L*. These genes, which are reported to be associated with growth, carcass, and internal organ traits of chickens, could play important roles in future genetic improvement efforts targeted at the chicken ecotypes of Ghana.

## Data Availability

The data presented in the study are deposited in the European Variation Archive (EVA) at EMBL-EBI under accession number PRJEB69292 (https://www.ebi.ac.uk/eva/?eva-study=PRJEB69292).
