# Supplementary material for: Genetic architecture of body weight, carcass, and internal organs traits of Ghanaian local chickens
Source: Front Genet. 2024 Mar 13;15:1297034. doi: 10.3389/fgene.2024.1297034 (PMC10976558; doi:10.3389/fgene.2024.1297034)
Supplement: Supplementary file 2 [file Image6.pdf]

K

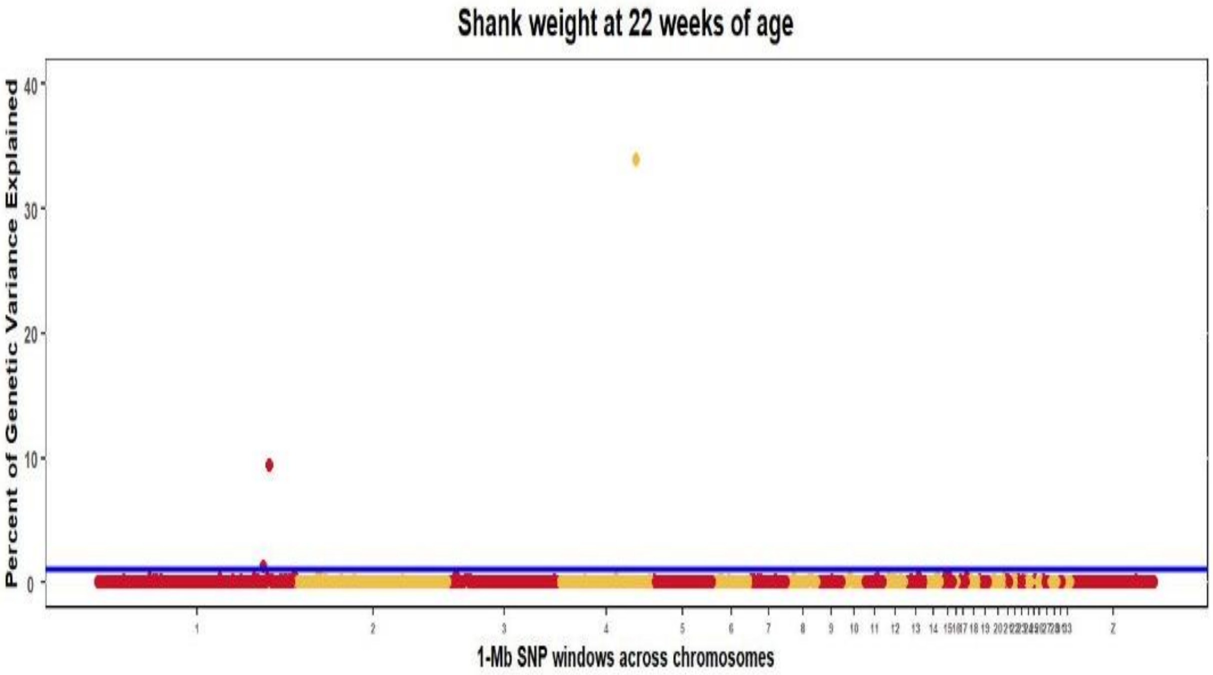

L

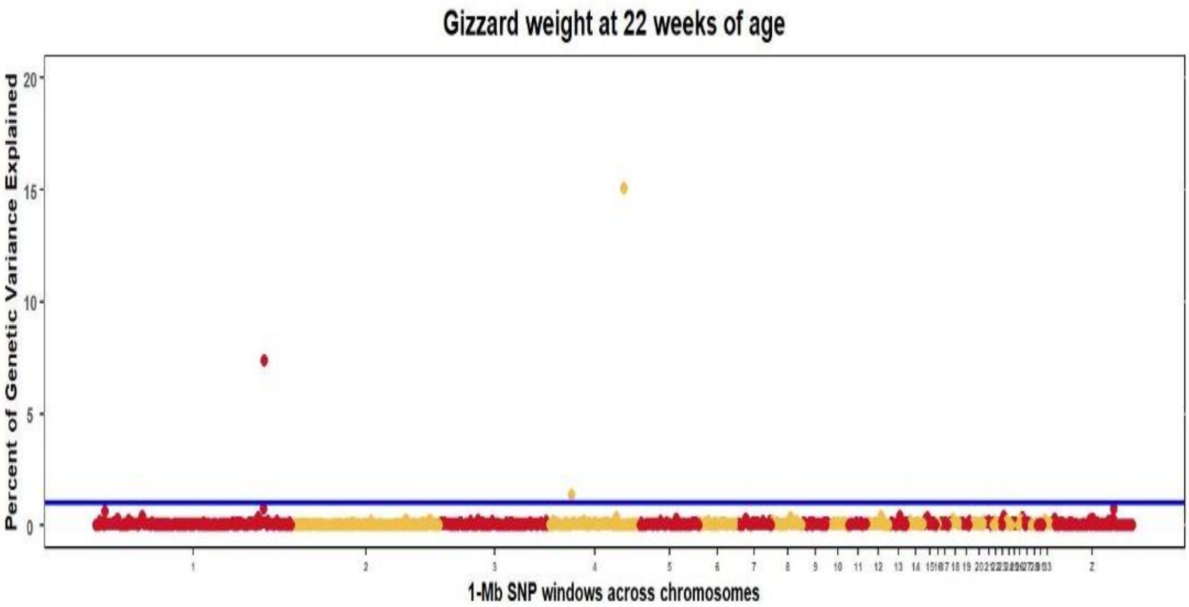

Supplementary Figure 6. Manhattan plot showing the genome-wide association results for Shank weight (K) and Gizzard (L). The plots show the percentage genetic variance explained by 1-Megabase (1-Mb) windows of SNPs across chromosomes (Model 1). The blue line indicates genome-wide significance at 1% genetic variance.
