# Supplementary material for: Genetic architecture of body weight, carcass, and internal organs traits of Ghanaian local chickens
Source: Front Genet. 2024 Mar 13;15:1297034. doi: 10.3389/fgene.2024.1297034 (PMC10976558; doi:10.3389/fgene.2024.1297034)
Supplement: Supplementary file 7 [file Image1.pdf]

A

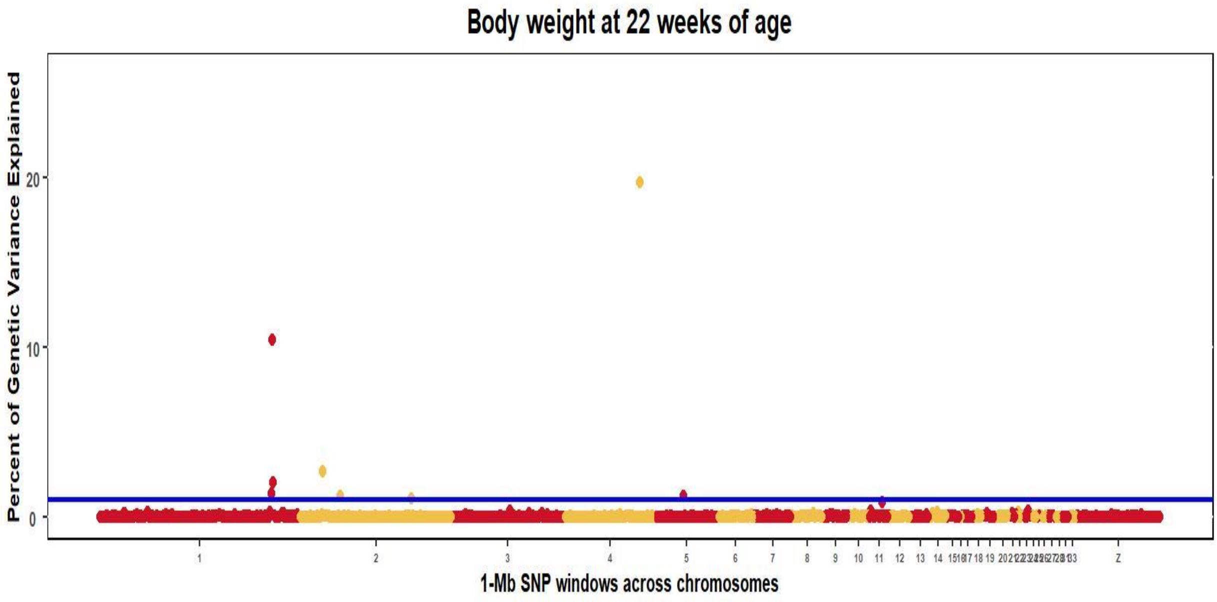

B

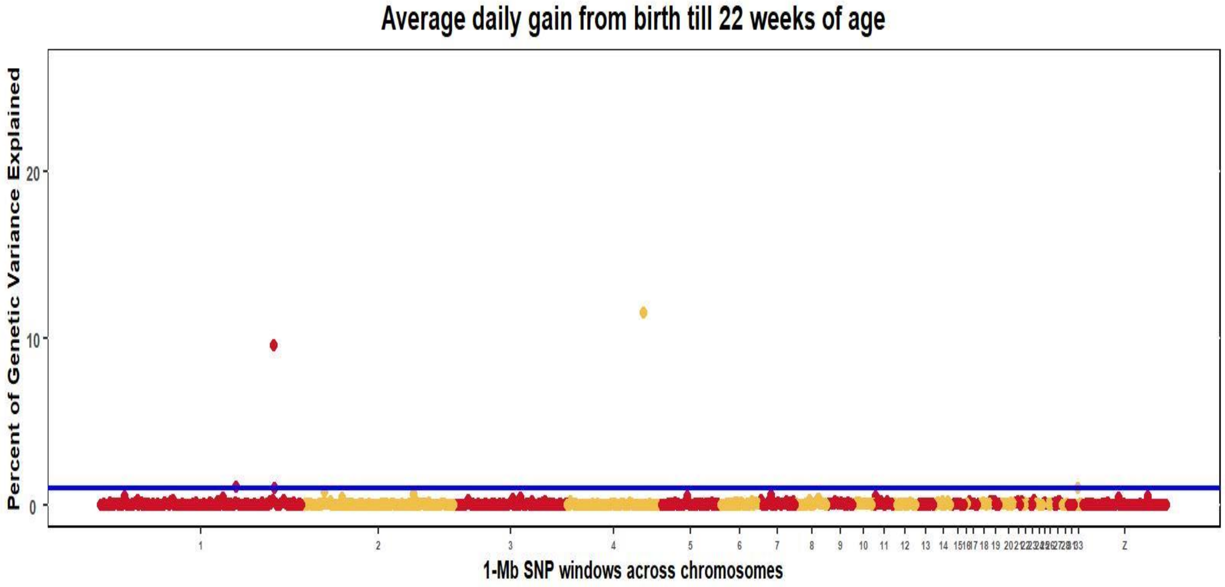

Supplementary Figure 1. Manhattan plots showing the genome-wide association results for Body weight (A) and Average daily Gain (B). The plots show the percentage genetic variance explained by 1-Megabase (1-Mb) windows of SNPs across chromosomes (Model 1). The blue line indicates genome-wide significance at 1% genetic variance.
